# Data to model risks for recolonizing wolves in Scandinavia through the integration of territory presence and human-driven mortalities

**DOI:** 10.1016/j.dib.2018.08.060

**Published:** 2018-08-28

**Authors:** Mariano R. Recio, Barbara Zimmermann, Camilla Wikenros, Andreas Zetterberg, Petter Wabakken, Håkan Sand

**Affiliations:** a Grimsö Wildlife Research Station, Department of Ecology, Swedish University of Agricultural Sciences, SE-73091 Riddarhyttan, Sweden; b Inland Norway University of Applied Sciences, Faculty of Applied Ecology, Agricultural Sciences and Biotechnology, Campus Evenstad, N-2480 Koppang, Norway

## Abstract

This dataset article describes the data and sources used to model risks for the recolonizing wolf (*Canis lupus*) in Sweden and Norway in the article “Integrated spatially-explicit models predict pervasive risks to recolonizing wolves in Scandinavia from human-driven mortality” (Recio et al., 2018). Presences on wolf territories were used to model the potential distribution of the species. Presences of human-driven mortalities including traffic collisions, culling (protective/defensive, and licensed hunting), and illegal killing (i.e. poaching) were used to model predictions on the distribution of these mortalities. Sources for the independent variables used for the models are also described.

**Specifications Table**

**Table:** 

Subject area	*Biology*
More specific subject area	*Ecology, conservation biology*
Type of data	*Text files*
How data was acquired	*Monitoring campaigns, animal telemetry (VHF and GPS), websites, governmental data*
Data format	*Analyzed*
Experimental factors	
Experimental features	
Data source location	*Sweden and Norway*
Data accessibility	Data is with this article.
	For the independent variables:
	http://land.copernicus.eu/pan-european/corine-land-cover/clc-2012
	www.scb.se
	www.ssb.no
	www.algdata.se
Related research article	M.R. Recio, B. Zimmermann, C. Wikenros, A. Zetterberg, P. Wabakken, H. Sand, Integrated spatially-explicit models predict pervasive risks to recolonizing wolves in Scandinavia from human-driven mortality. Biol Conserv, 226 (2018), pp. 111–119. [1]

**Value of the data**

● A compilation of the wolf monitoring reports used to produce the distribution dataset on wolf territories in Scandinavia is included to inform further studies on wolf expansion.
● These data show the presences of four causes of human-driven mortality of wolves in Scandinavia, which is of relevance for further research on the distribution and occurrence of these mortalities.
● The descriptions and sources of the data related to the independent variables used on the models can facilitate the search for these data to further research.
● These data provide a benchmark for further studies on the impact of human-driven mortality on wolf population and expansion in Scandinavia.

## 1 Data

**Fig. 1:**
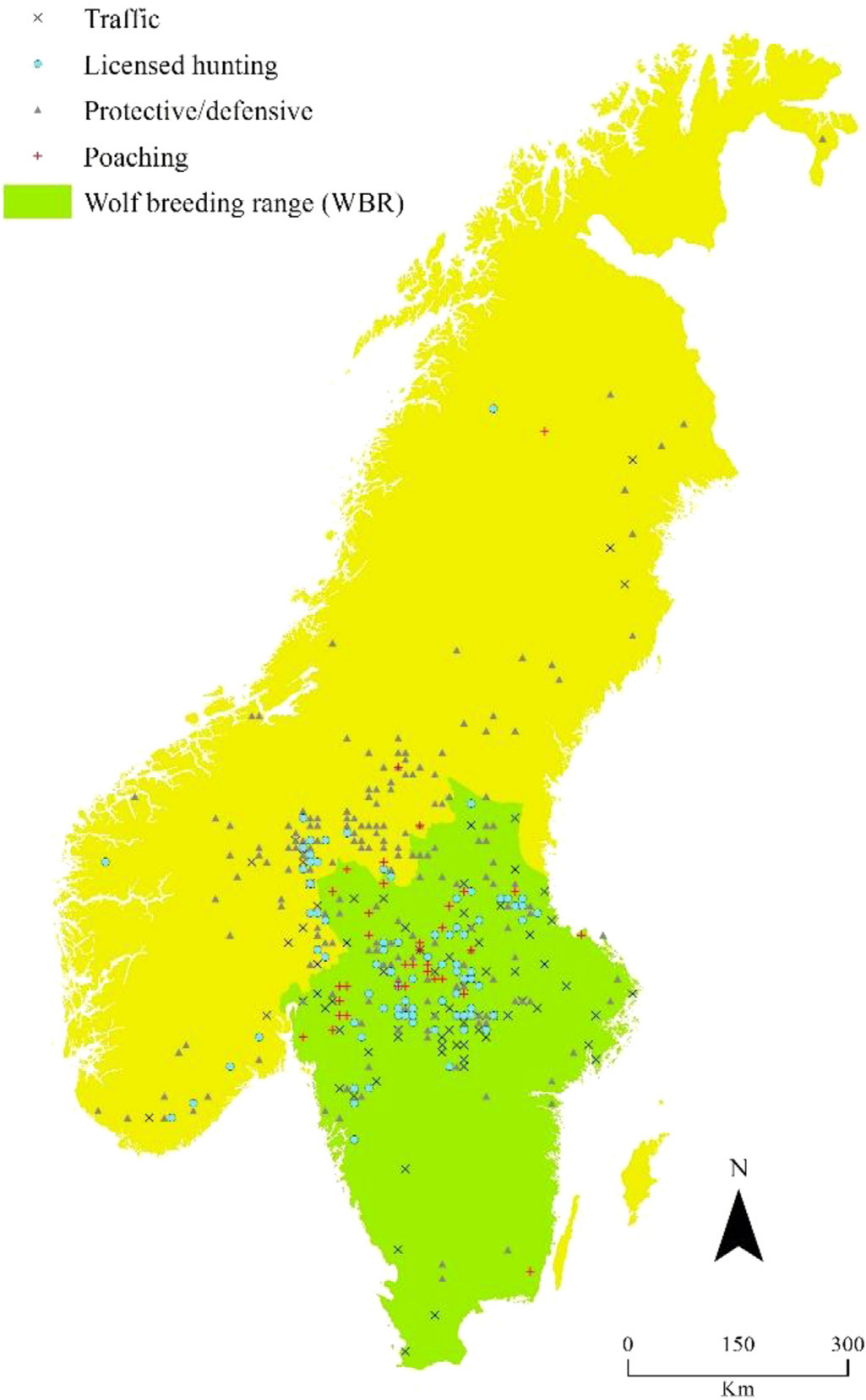
Points depicting the grid-cells of mortality presence used in the different mortality models (traffic, license hunting, protective/defensive, and poaching mortality) in Maxent.

For the species distribution models (SDM) on the distribution of wolf territories [1], we identified wolf territory presence in Sweden and Norway (hereafter Scandinavia) from published annual monitoring reports (see the full list in Supplementary material). These annual reports have compiled the information on monitored wolf presence and population distribution in Scandinavia since 1978. The monitoring campaigns are conducted by regional and national authorities in collaboration with NGOs and the general public combining snow tracking, DNA sampling (faeces, urine), and dead individuals. Data from > 160 tracked wolves during 1998–2016 using very high frequency (VHF) and global positioning system (GPS) collars are also included in the monitoring reports. Wolf territories are estimated in these reports using minimum convex polygon (MCP) methods as explained in Recio et al. (2018). For models on human-driven wolf mortality [1], we used the locations of reported collisions involving cars or trains (*N* = 74) since 1999, as well as our compilation of poaching, protective/defensive, and licensed hunting events (Fig. 1).

The independent variables used to model wolf presence and human-driven mortality presence included assorted land cover variables, transport infrastructures (road and railways), human population density, number of sheep farms, brown bear (*Ursus arctos*) presence, presence of wolf breeding range (WBR) or not, and surrogates of prey abundance. We compiled the landscape variables from CORINE Land cover 2012, 250 m resolution [2]. Transport infrastructures were extracted from the National Road Database (NVDB) at the Swedish Transport Administration status 2014 and from digital maps of the Norwegian Mapping Authorities. We collected human population density (number of inhabitants/km^2^) from Statistics Sweden [3], and Statistics Norway [4]. Numbers on sheep farms were compiled from the dataset used in Frank et al. [5]. Brown bear distribution in Scandinavia was a categorical variable as reported by Boitani et al. [6]. For estimating prey abundances from surrogates, we used the number of moose shot in the moose management units in Sweden and the municipalities in Norway during the 2012 to 2015 hunting seasons [4], [7], [8]. Similar data were obtained for roe deer (*Capreolus capreolus*), red deer (*Cervus elaphus*), fallow deer (*Dama dama*), and wild boar (*Sus scrofa*) within municipalities in both countries from the Swedish Association for Hunting and Wildlife Management and Statistics Norway [4].

## 2 Experimental design, materials and methods

We used ArcGIS software (Redlands, CA) to quantify and process all the variables and digital layers, and to produce a 10 × 10 km grid as the sampling unit. To produce the presence data to model the distribution of wolf territories in Maxent [9], we randomly selected centroids of the 10 × 10 km grids contained in each territory as minimum convex polygons (MCP) produced from the annual monitoring campaigns, but each centroid separated by at least 13 km, which guaranteed no consecutive cells were selected to reduce spatial autocorrelation (see Fig. 1. in [1]). A total of 396 centroids were produced as territory presence points and 20% were randomly taken out for independent tests on model accuracy. This characterization of territory presences can concentrate a larger number of non-independent points in territories estimated from individuals with VHF/GPS collars (representing real outermost boundaries of territories) than in those delineated from DNA-sampling and snow tracks (often smaller and estimated from a small sample of locations). Therefore, we produced a sampling bias raster for the background point selection in Maxent accounting for this sampling difference and weighted the more isolated points [10]. This raster was created using a Gaussian kernel density of sampling localities in the SDMToolbox for ArcGIS [11], with a sampling bias distance equal to the length of the largest territory.

To model the mortality caused by traffic in Maxent, we identified for each 10 × 10 km grid the presence of traffic mortality from the locations of reported collisions. The number of grid-cells with the presence of wolf hunted and killed varied between cases (*N* = 35, *N* = 202, *N* = 100 for poaching, protective/defensive, and licensed hunting, respectively, Fig. 1). As for the SDM, we also created a sample bias for each hunting model using the same criteria. The motivation was because the identified hunting events were not the result of a sampling design but caused by management or poaching decisions derived from the human-wolf conflict, which is *a priori* more likely to occur with more intense wolf presence.

For the independent variables, we split land cover as natural and anthropogenic land cover. Natural land cover variables included forest and other natural non-forest vegetation; the latter resulted after merging the CORINE class shrublands, open natural landscapes, and inland wetlands. Anthropogenic land cover variables were added to account for the indirect impact of the human transformation of the landscape on the occurrence of wolf territories and included agricultural areas and other anthropogenic artificial surfaces. Land cover variables were calculated as the percentage of cover in each 10 × 10 km grid-cell. We calculated the total density of roads and railways transport infrastructures as the number of kilometres per grid-cell, human population density as the number of inhabitants/km^2^ as a surrogate of the grade of humanization. We also used the number of sheep farms per grid-cell. Brown bear distribution was a categorical variable depicting the three presence levels: population reproductive cores, sporadic presence, and absence. We delineated the WBR area as the result of merging the area to the south of the reindeer husbandry area in Sweden with the ‘Norwegian wolf zone’. For each prey species, we calculated the number animals shot per square kilometre within each grid-cell as a surrogate of abundance.
