# Supplementary material for: Data to model risks for recolonizing wolves in Scandinavia through the integration of territory presence and human-driven mortalities
Source: Data Brief. 2018 Aug 28;20:686–90. doi: 10.1016/j.dib.2018.08.060 (PMC6129723; doi:10.1016/j.dib.2018.08.060)
Supplement: Supplementary file 1 — Supplementary material [file mmc1.docx]

Dear Editorial Office,

I am pleased to submit the original data article entitled “Data to model risks for recolonizing wolves in Scandinavia through the integration of territory presence and human-driven mortalities” by Mariano R. Recio et al.

We declare that:

- The work is all original research carried out by the authors.
- All authors agree with the contents of the manuscript and its submission to the journal.
- The manuscript is not being considered for publication elsewhere.
- Any research in the paper not carried out by the authors are fully acknowledged in the manuscript.
- All sources of funding are acknowledged in the manuscript, and no direct financial benefits could result from publication.
- All appropriate ethics and other approvals were obtained for the research.

Sincerely,

Mariano R. Recio
