# Supplementary material for: Data to model risks for recolonizing wolves in Scandinavia through the integration of territory presence and human-driven mortalities
Source: Data Brief. 2018 Aug 28;20:686–90. doi: 10.1016/j.dib.2018.08.060 (PMC6129723; doi:10.1016/j.dib.2018.08.060)
Supplement: Supplementary file 2 — Supplementary material [file mmc2.docx]

***Wolf monitoring in Scandinavia***

Wolf presence and population distribution in Scandinavia have been monitored by regional and national authorities in collaboration with NGOs and the general public since 1978 (see also the list of annual reports below) combining snow tracking, DNA sampling (feces, urine), and dead individuals. Data from >160 tracked wolves during 1998-2016 using very high frequency (VHF) and global positioning system (GPS) collars have also been included the monitoring reports.

***List of annual reports***

Wabakken, P., Aronson, Å., Sand, H., Steinset, O.K., & Kojola, I. (1999). Ulv i Skandinavia: Statusrapport for vinteren 1998-99. Høgskolen i Hedmark, rapport nr. 19 - 99. 46 s.

Wabakken, P., Aronson, Å., Sand, H., Steinset, O.K., & Kojola, I. (2000). Varg i Skandinavien: Statusrapport för vintern 1999-2000. Høgskolen i Hedmark. Oppdragsrapport nr. 2 - 2000. 70 s.

Aronson, Å., Wabakken, P., Sand, H., Steinset, O.K., & Kojola, I. (2001). Varg i Skandinavien: Statusrapport for vintern 2000/2001. Høgskolen i Hedmark, Oppdragsrapport nr. 2 - 2001. 53 s.

Wabakken, P., Aronson, Å., Sand, H., Steinset, O.K., & Kojola, I. (2002). Ulv i Skandinavia: Statusrapport for vinteren 2001-2002. Høgskolen i Hedmark, Oppdragsrapport nr. 2 - 2002. 40 s.

Wabakken, P., Aronson, Å., Sand, H., Rønning, H., & Kojola, I. (2004). Ulv i Skandinavia: Statusrapport for vinteren 2002-2003. Høgskolen i Hedmark, Oppdragsrapport nr. 2 - 2004. 46 s.

Wabakken, P., Aronson, Å., Sand, H., Strømseth, T., & Kojola, I. (2004). Ulv i Skandinavia: Statusrapport for vinteren 2003-2004. Høgskolen i Hedmark, Oppdragsrapport nr. 5 - 2004. 41 s.

Wabakken, P., Aronson, Å., Strømseth, T.H., Sand, H., & Kojola, I. (2005). Ulv i Skandinavia: Statusrapport for vinteren 2004-2005. Høgskolen i Hedmark, Oppdragsrapport nr. 6 - 2005. 47 s.

Wabakken, P., Aronson, Å., Strømseth, T.H., Sand, H., Svensson, L., & Kojola I. (2006). Ulv i Skandinavia: Statusrapport for vinteren 2005-2006. Høgskolen i Hedmark, Oppdragsrapport nr. 2 - 2006. 42 s.

Wabakken, P., Aronson, Å., Strømseth, T.H., Sand, H., Svensson, L., & Kojola I. (2007). Ulv i Skandinavia: Statusrapport for vinteren 2006-2007. Høgskolen i Hedmark, Oppdragsrapport nr. 6 - 2007. 49 s.

Wabakken, P., Aronson, Å., Strømseth, T.H., Sand, H., Svensson, L., & Kojola I. (2008). Ulv i Skandinavia: Statusrapport for vinteren 2007-2008. Høgskolen i Hedmark, Oppdragsrapport nr. 6 - 2008. 53 s.

Wabakken, P., Aronson, Å., Strømseth, T.H., Sand, H., Maartmann, E., Svensson, L., & Kojola, I. (2009). Ulv i Skandinavia: Statusrapport for vinteren 2008-2009. Høgskolen i Hedmark, Oppdragsrapport nr. 6 - 2009. 51 s.

Wabakken, P., Aronson, Å., Strømseth, T.H., Sand, H., Maartmann, E., Svensson, L., Flagstad, Ø., Hedmark, E., Liberg, O., & Kojola, I. (2010). Ulv i Skandinavia: Statusrapport for vinteren 2009-2010. Høgskolen i Hedmark, Oppdragsrapport nr. 4 - 2010. 57 s.

Wabakken, P., Aronson, Å., Strømseth, T.H., Sand, H., Maartmann, E., Svensson, L., Åkesson, M., Flagstad, Ø., Liberg, O., & Kojola, I. (2011). Ulv i Skandinavia: Statusrapport for vinteren 2010-2011. Høgskolen i Hedmark, Oppdragsrapport nr. 1 - 2011. 60 s.

Wabakken, P., Svensson, L., Kojola, I., Maartmann, E., Strømseth, T.H., Flagstad, Ø., Åkesson, M., & Zetterberg, A. (2012). Ulv i Skandinavia og Finland: Slutrapport for bestandsovervåking av ulv vinteren 2011-2012. Høgskolen i Hedmark, Oppdragsrapport nr. 5 - 2012. 46 s.

Wabakken, P., Svensson, L., Kojola, I., Maartmann, E., Strømseth, T.H., Flagstad, Ø., Åkesson, M., & Zetterberg, A. (2013). Ulv i Skandinavia og Finland: Slutrapport for bestandsovervåking av ulv vinteren 2012-2013. Høgskolen i Hedmark, Oppdragsrapport nr. 5 - 2013. 34 s.

Anon. 2015. Bestandsovervåking av ulv vinteren 2014-2015. Bestandsstatus for store rovdyr i Skandinavia. Redaktion: Wabakken, P., Svensson, L., Maartmann, E., Flagstad, Ø., & Åkesson, M.. Rovdata, Rapport nr. 1 – 2015. 52 s.

Wabakken, P., Svensson, L., Kojola, I., Maartmann, E., Strømseth, T.H., Flagstad, Ø., Åkesson, M. (2014). Ulv i Skandinavia og Finland: Slutrapport for bestandsovervåking av ulv vinteren 2013-2014. Høgskolen i Hedmark, Oppdragsrapport nr. 11 - 2014. 40 s.

Wabakken, P., Svensson, L., Maartmann, E., Åkesson, M., & Flagstad, Ø. (2016). Bestandsovervåking av ulv vinteren 2015-2016/Inventering av varg vintern 2015-2016. Bestandsstatus for store rovdyr i Skandinavia/Beståndsstatus för stora rovdjur i Skandinavien 1-2016. 49 s.
